# Hormonal Modulation of Ventricular Repolarization Dynamics During the Menstrual Cycle

**DOI:** 10.1111/anec.70163

**Published:** 2026-02-09

**Authors:** Mucahit Yetim, Macit Kalçık

**Affiliations:** ^1^ Department of Cardiology, Faculty of Medicine Hitit University Corum Turkey

To the Editor,

1

We read with interest the article by San et al. examining the association between sex hormone fluctuations and ventricular repolarization dynamics across the menstrual cycle in women treated with QT‐prolonging drugs (San et al. 2026). The authors should be acknowledged for conducting a prospective study with repeated ECG recordings and parallel hormone assessments, an approach that is technically demanding and rarely implemented. The focus on QT‐Apex as an alternative marker of repolarization is also noteworthy. Nevertheless, several aspects of study design and interpretation warrant closer scrutiny.

A primary limitation is the small sample size and marked heterogeneity between the treatment and control groups. Women receiving dofetilide or sotalol were significantly older and had a substantially higher burden of cardiovascular comorbidities than controls, factors independently known to influence repolarization parameters (Lehmann et al. 1996). Although mixed‐effects models were used, residual confounding related to age, structural heart disease, and atrial arrhythmia history cannot be excluded. This imbalance complicates attribution of observed QT‐Apex changes solely to hormonal variation.

Second, the clinical significance of QT‐Apex modulation remains uncertain. While prior work suggests that QT‐Apex may capture early repolarization abnormalities, its relationship with hard arrhythmic outcomes is far less established than that of QTc (Haapalahti et al. 2011). The study reports statistically significant correlations measured in milliseconds, yet it remains unclear whether these modest changes translate into a meaningful difference in torsade de pointes risk. Without outcome data or established thresholds, interpretation should remain cautious.

Third, hormone assessment relied on single‐day salivary measurements for each menstrual phase, which may not accurately reflect integrated hormonal exposure over the seven‐day ECG recording periods. Sex hormone levels are known to exhibit substantial intra‐phase variability, and single time‐point sampling risks misclassification (Sedlak et al. 2012). More frequent or averaged measurements could have strengthened the biological plausibility of the reported associations.

Finally, extrapolation of these findings to broader clinical practice appears premature. The study population was limited to women treated with two class III antiarrhythmic agents, and results may not apply to other QT‐prolonging drugs commonly prescribed in clinical practice. Prior literature demonstrates that drug‐specific ion channel interactions and sex‐related differences vary considerably across agents (Zareba 2007). Larger, outcome‐oriented studies across multiple drug classes are required before hormonal status can be incorporated into arrhythmic risk stratification.

Sincerely,

## Author Contributions

All of the authors contributed planning, writing, and revision.

## Funding

The authors have nothing to report.

## Conflicts of Interest

The authors declare no conflicts of interest.

## Data Availability

The authors have nothing to report.
